# Supplementary material for: New Antimalarial and Antimicrobial Tryptamine Derivatives from the Marine Sponge Fascaplysinopsis reticulata
Source: Mar Drugs. 2019 Mar 15;17(3):167. doi: 10.3390/md17030167 (PMC6471642; doi:10.3390/md17030167)

Supporting information

New Antimalarial and Antimicrobial Tryptamine Derivatives from the Marine Sponge *Fascaplysinopsis reticulata*

Pierre-Eric Campos[1], Emmanuel Pichon[1], Céline Moriou[2], Patricia Clerc[1], Rozenn Trépos[3], Michel Frederich[4], Nicole De Voogd[5], Claire Helio[3], Anne Gauvin-Bialecki[1*], and Ali Al-Mourabit[2]

[1] Laboratoire de Chimie des Substances Naturelles et des Sciences des Aliments, Faculté des Sciences et Technologies, Université de La Réunion, 15 Avenue René Cassin, CS 92003, 97744 Saint-Denis Cedex 9, La Réunion, France

[2] Institut de Chimie des Substances Naturelles, CNRS UPR 2301, Univ. Paris-Sud, Université Paris-Saclay, 1, av. de la Terrasse, 91198 Gif-sur-Yvette, France

[3] Univ Brest, CNRS, IRD, Ifremer, LEMAR, F-29280 Plouzane, France

[4] Laboratory of Pharmacognosy, Center for Interdisciplinary Research on Medicines, CIRM, University of Liège B36, 4000 Liège, Belgium

[5] Naturalis Biodiversity Center, Darwinweg 2, 2333 CR Leiden, Netherlands

Figure S1: HRMS spectrum for 6,6'-bis-(debromo)-gelliusine F (**1**) 4
Figure S2: ^{1}H NMR (300 MHz, MeOD) spectrum for 6,6'-bis-(debromo)-gelliusine F (**1**) 5
Figure S3: ^{13}C NMR (300 MHz, MeOD) spectrum for 6,6'-bis-(debromo)-gelliusine F (**1**) 6
Figure S4: ^{1}H-^{1}H COSY NMR (300 MHz, MeOD) spectrum for 6,6'-bis-(debromo)-gelliusine F (**1**)........ 7
Figure S5: HSQC NMR (300 MHz, MeOD) spectrum for 6,6'-bis-(debromo)-gelliusine F (**1**)........ 8
Figure S6: ^{1}H-^{13}C HMBC NMR (300 MHz, MeOD) spectrum for 6,6'-bis-(debromo)-gelliusine F (**1**) 9
Figure S7: HRMS spectrum for 6-bromo-8,1'-dihydro-isoplysin A (**2**) 10
Figure S8: ^{1}H NMR (300 MHz, MeOD) spectrum for 6-bromo-8,1'-dihydro-isoplysin A (**2**) 11
Figure S9: ^{13}C NMR (300 MHz, MeOD) spectrum for 6-bromo-8,1'-dihydro-isoplysin A (**2**) 12
Figure S10: ^{1}H-^{1}H COSY NMR (300 MHz, MeOD) spectrum for 6-bromo-8,1'-dihydro-isoplysin A (**2**). 13
Figure S11: HSQC NMR (300 MHz, MeOD) spectrum for 6-bromo-8,1'-dihydro-isoplysin A (**2**)........ 14
Figure S12: ^{1}H-^{13}C HMBC NMR (300 MHz, MeOD) spectrum for 6-bromo-8,1'-dihydro-isoplysin A (**2**) 15
Figure S13: HRMS spectrum for 5,6-dibromo-8,1'-dihydro-isoplysin A (**3**) 16
Figure S14: ^{1}H NMR (600 MHz, MeOD) spectrum for 5,6-dibromo-8,1'-dihydro-isoplysin A (**3**) 17
Figure S15: ^{13}C NMR (600 MHz, MeOD) spectrum for 5,6-dibromo-8,1'-dihydro-isoplysin A (**3**) 18
Figure S16: ^{1}H NMR (600 MHz, MeOD) spectrum for 8-oxo-tryptamine (**4**) 19
Figure S17: ^{13}C NMR (600 MHz, MeOD) spectrum for 8-oxo-tryptamine (**4**) 20
Figure S18: ^{1}H NMR (300 MHz, MeOD) spectrum for tryptamine (**5**)........ 21
Figure S19: ^{13}C NMR (300 MHz, MeOD) spectrum for tryptamine (**5**)........ 22
Figure S20: ^{1}H NMR (500 MHz, DMSO) spectrum for (E)-6-bromo-2'-demethyl-3'-*N*-methylaplysinopsine (**6**) and (Z)-6-bromo-2'-demethyl-3'-*N*-methylaplysinopsine (**7**)........ 23
Figure S21: ^{13}C NMR (500 MHz, MeOD) spectrum for (*E*)-6-bromo-2'-demethyl-3'-*N*-methylaplysinopsine (**6**) and (*Z*)-6-bromo-2'-demethyl-3'-*N*-methylaplysinopsine (**7**)........ 24

S0. Observed data for known compounds.

8-oxo-tryptamine (**4**): red oil, ^{1}H and ^{13}C NMR data see **Supporting Information**; HRESIMS *m/z* 175.0993 $[M + H]^+$ (calcd for $C_{10}H_{11}N_2O$, 175.0871).

Tryptamine (**5**): white powder, ^{1}H and ^{13}C NMR data see **Supporting Information**; HRESIMS *m/z* 161.1219 $[M + H]^+$ (calc for $C_{10}H_{13}N_2$, 161.1079).

(E)-6-bromo-2′-demethyl-3′-N-methylaplysinopsine (**6**) : yellow oil, ^{1}H and ^{13}C data see **Supporting Information**; HRESIMS *m/z* 335.0325 $[M + H]^+$ (calc for $C_{14}H_{14}N_4O^{81}Br$, 335.0331).

(Z)-6-bromo-2′-demethyl-3′-N-methylaplysinopsine (**7**) : yellow oil, ^{1}H and ^{13}C data see **Supporting Information**; HRESIMS *m/z* 335.0325 $[M + H]^+$ (calc for $C_{14}H_{14}N_4O^{81}Br$, 335.0331).

Figure S1: HRMS spectrum for 6,6'-bis-(debromo)-gelliusine F (**1**)

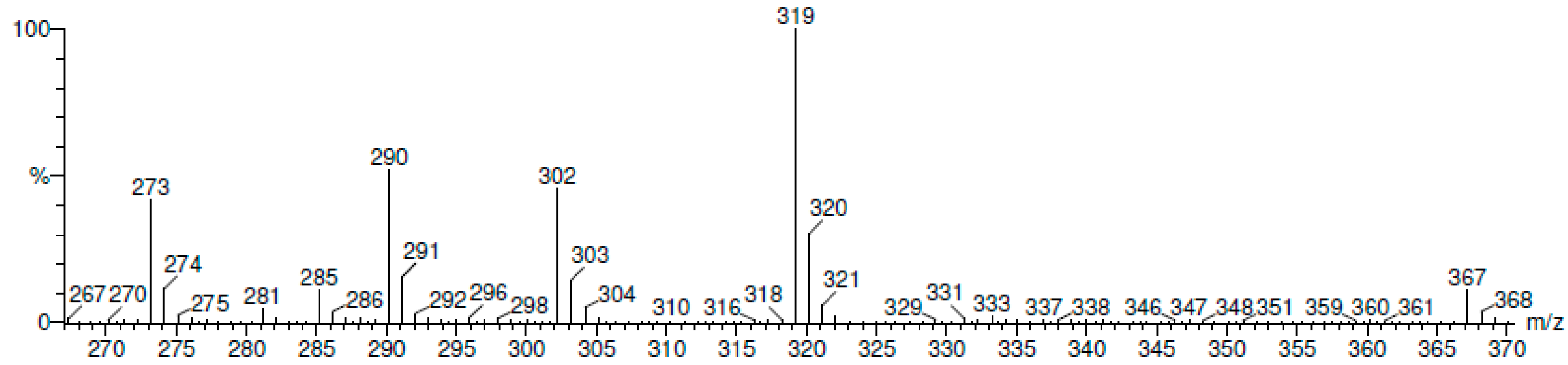

Minimum:				-1.5			
Maximum:		15.0	10.0	50.0			
Mass	Calc. Mass	mDa	PPM	DBE	i-FIT	i-FIT (Norm)	Formula
319.2013	319.2062	-4.9	-15.4	10.5	38.9	0.7	C23 H27 O
	319.1923	9.0	28.2	11.5	40.1	2.0	C20 H23 N4
	319.1909	10.4	32.6	6.5	40.6	2.5	C19 H27 O4
	319.2022	-0.9	-2.8	6.5	40.9	2.8	C18 H27 N2 O3
	319.2134	-12.1	-37.9	6.5	41.2	3.0	C17 H27 N4 O2
	319.2121	-10.8	-33.8	1.5	41.7	3.5	C16 H31 O6
	319.1882	13.1	41.0	7.5	41.7	3.6	C15 H23 N6 O2
	319.1995	1.8	5.6	7.5	41.9	3.8	C14 H23 N8 O
	319.1869	14.4	45.1	2.5	42.1	4.0	C14 H27 N2 O6
	319.1981	3.2	10.0	2.5	42.3	4.2	C13 H27 N4 O5
	319.2107	-9.4	-29.4	7.5	42.0	3.9	C13 H23 N10
	319.2094	-8.1	-25.4	2.5	42.5	4.3	C12 H27 N6 O4
	319.1955	5.8	18.2	3.5	42.9	4.8	C9 H23 N10 O3
	319.2067	-5.4	-16.9	3.5	43.1	4.9	C8 H23 N12 O2
	319.1941	7.2	22.6	-1.5	43.3	5.2	C8 H27 N6 O7
	319.2054	-4.1	-12.8	-1.5	43.5	5.3	C7 H27 N8 O6
	319.1928	8.5	26.6	4.5	43.4	5.3	C5 H19 N16 O
	319.2040	-2.7	-8.5	4.5	43.3	5.2	C4 H19 N18
	319.1914	9.9	31.0	-0.5	43.8	5.7	C4 H23 N12 O5
	319.2027	-1.4	-4.4	-0.5	43.9	5.8	C3 H23 N14 O4
	319.2139	-12.6	-39.5	-0.5	44.0	5.9	C2 H23 N16 O3
	319.1888	12.5	39.2	0.5	44.2	6.1	H19 N18 O3

Figure S2: ^{1}H NMR (300 MHz, MeOD) spectrum for 6,6'-bis-(debromo)-gelliusine F (**1**)

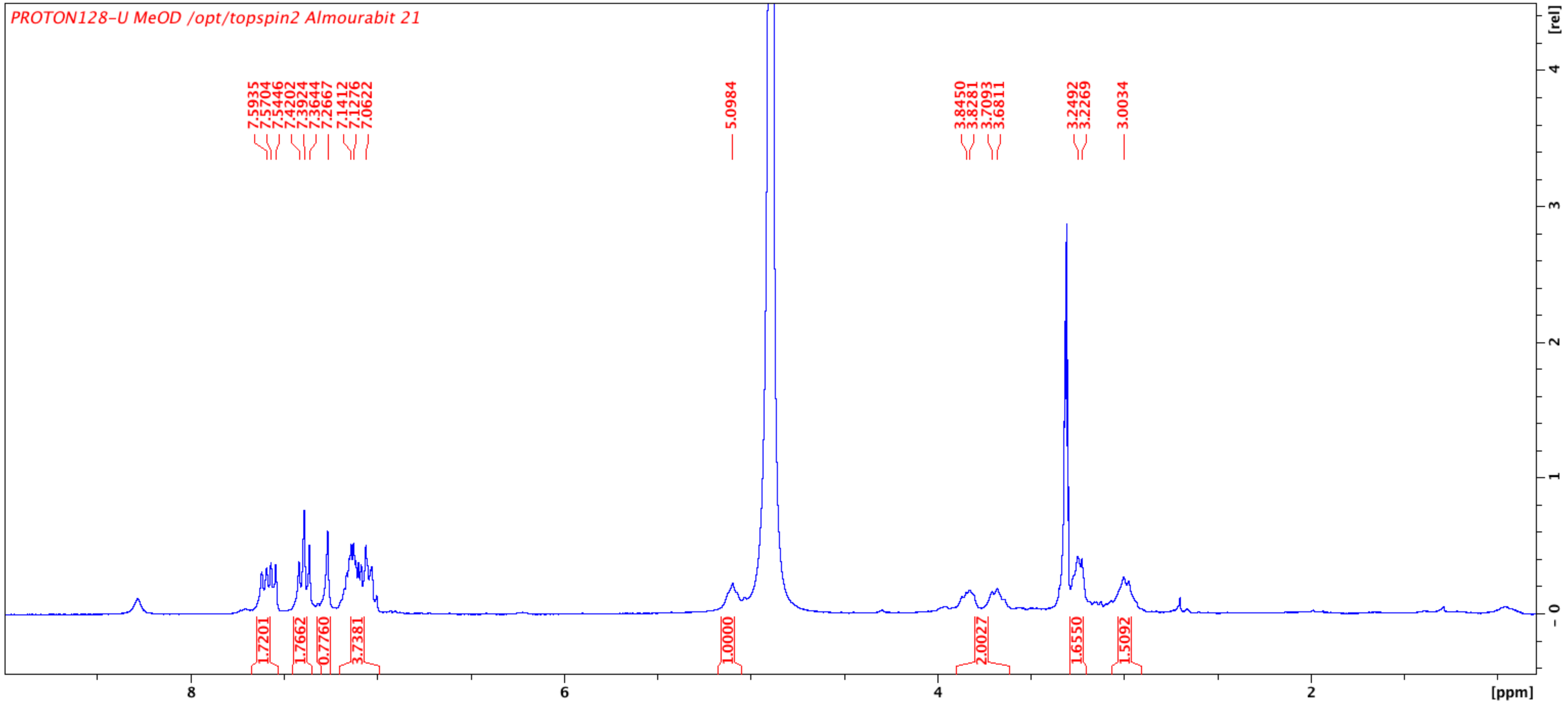

Figure S3: ^{13}C NMR (300 MHz, MeOD) spectrum for 6,6'-bis-(debromo)-gelliusine F (**1**)

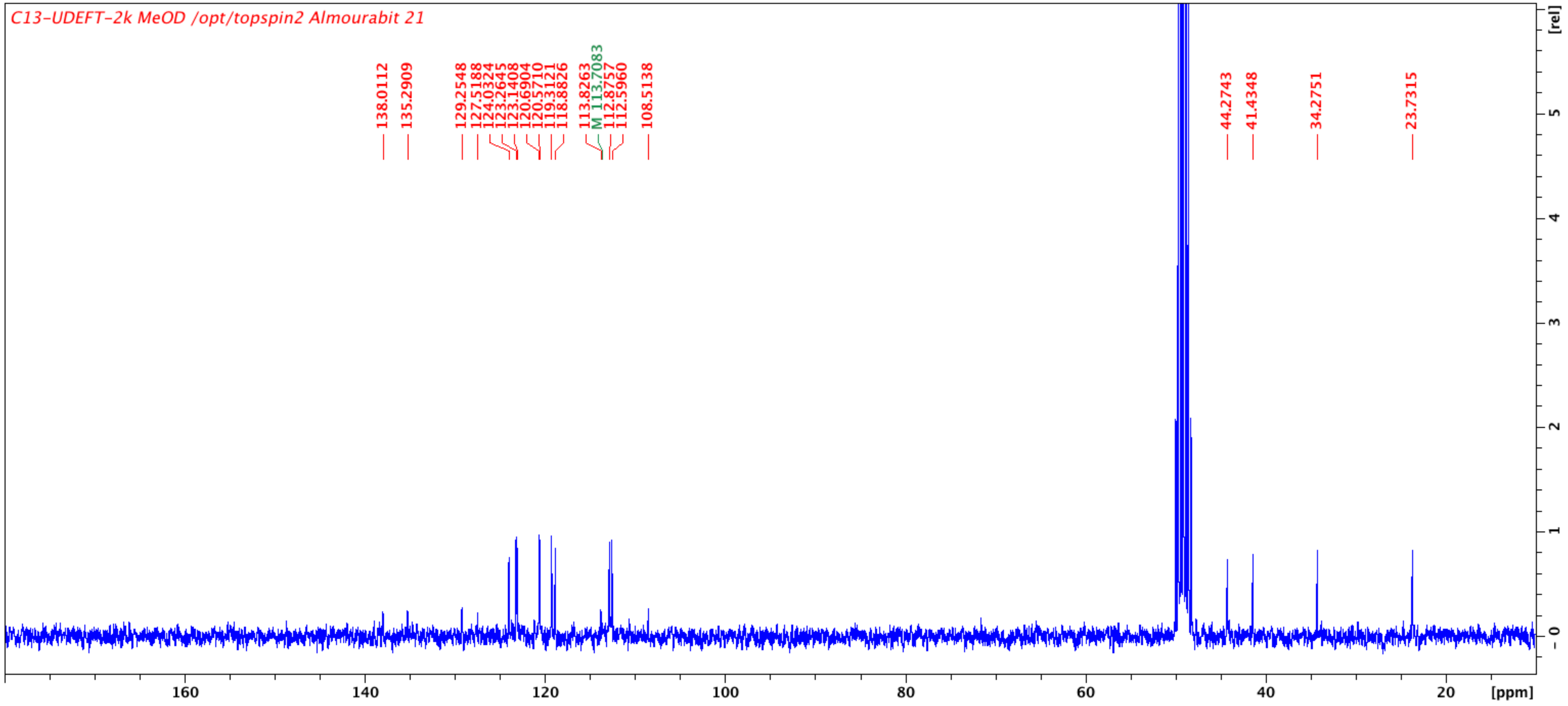

Figure S4: ^{1}H-^{1}H COSY NMR (300 MHz, MeOD) spectrum for 6,6'-bis-(debromo)-gelliusine F (**1**)

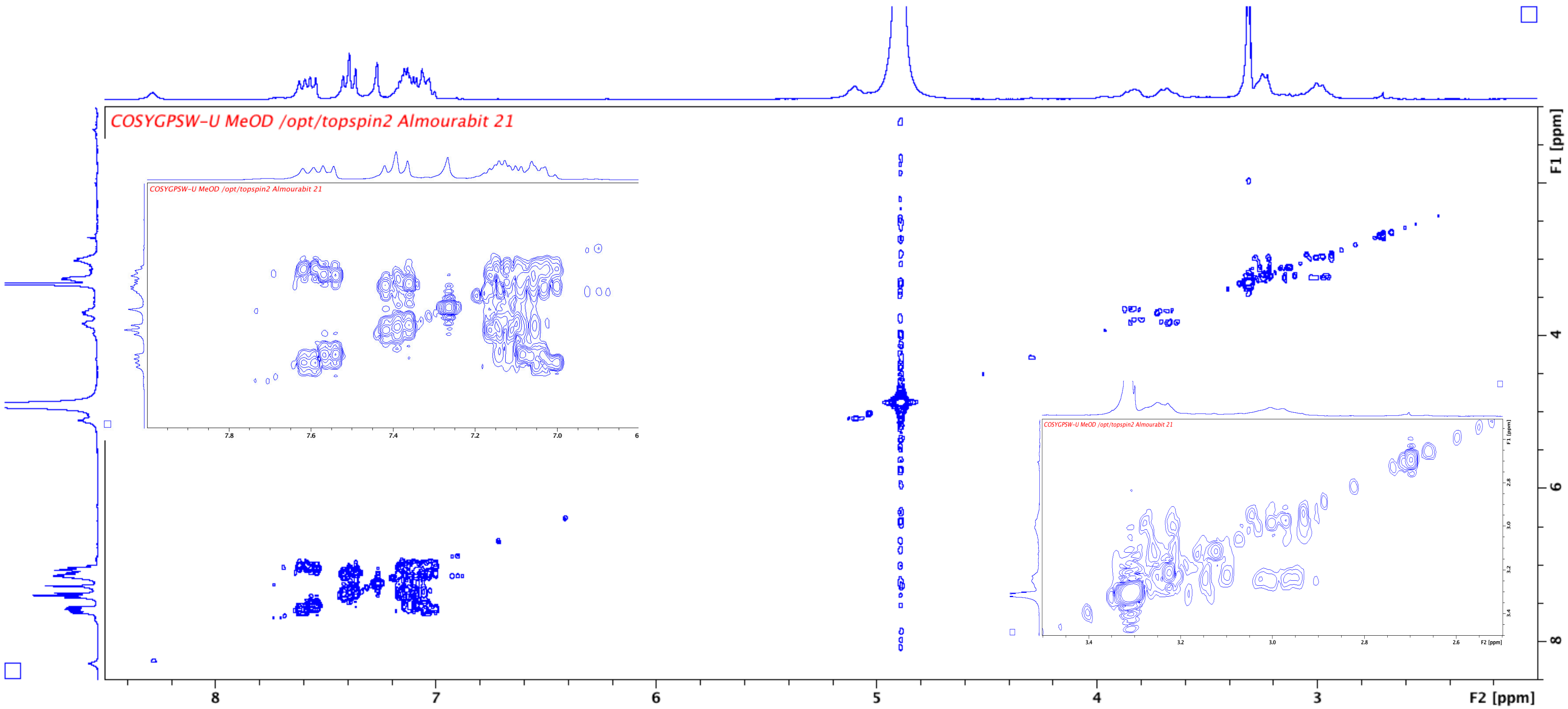

Figure S5: HSQC NMR (300 MHz, MeOD) spectrum for 6,6'-bis-(debromo)-gelliusine F (**1**)

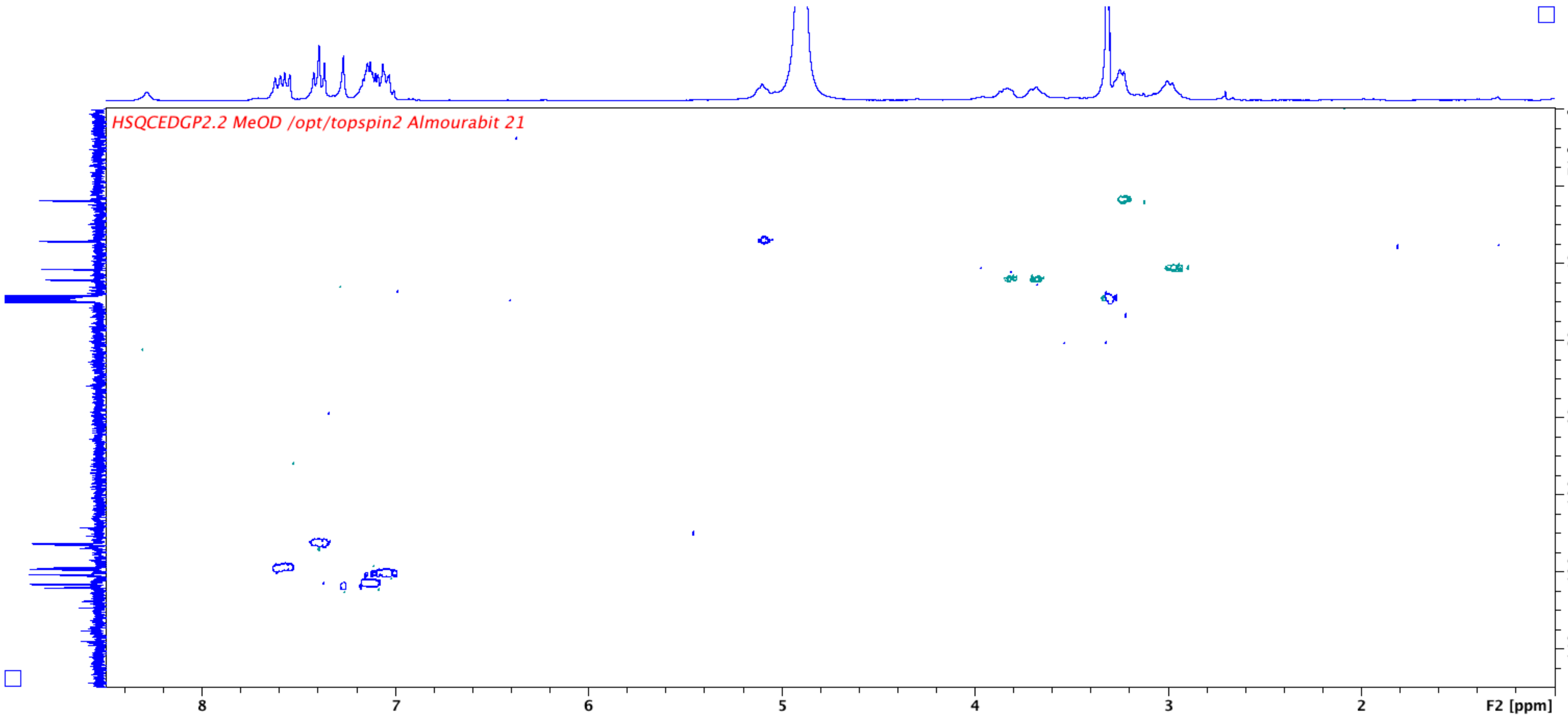

Figure S6: ^{1}H-^{13}C HMBC NMR (300 MHz, MeOD) spectrum for 6,6'-bis-(debromo)-gelliusine F (**1**)

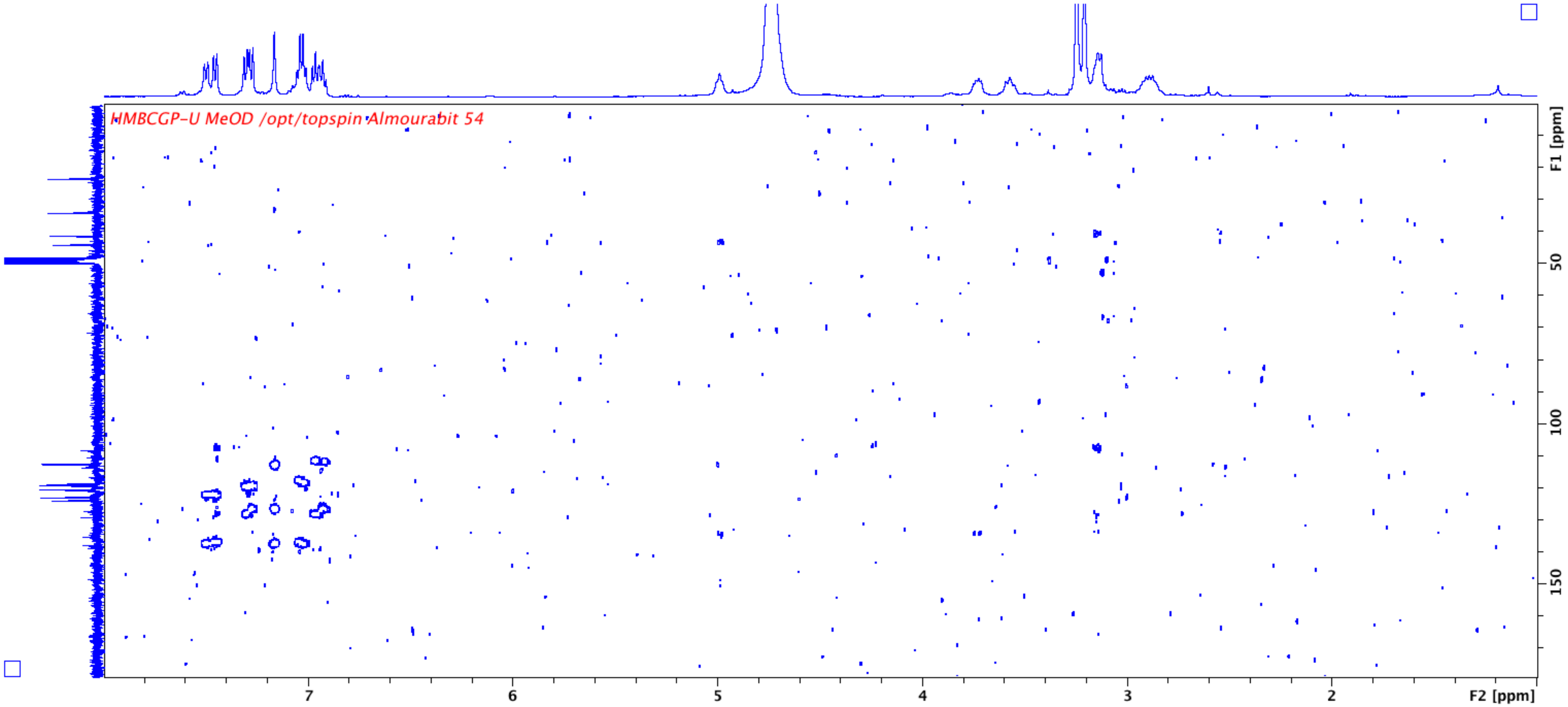

Figure S7: HRMS spectrum for 6-bromo-8,1'-dihydro-isoplysin A (**2**)

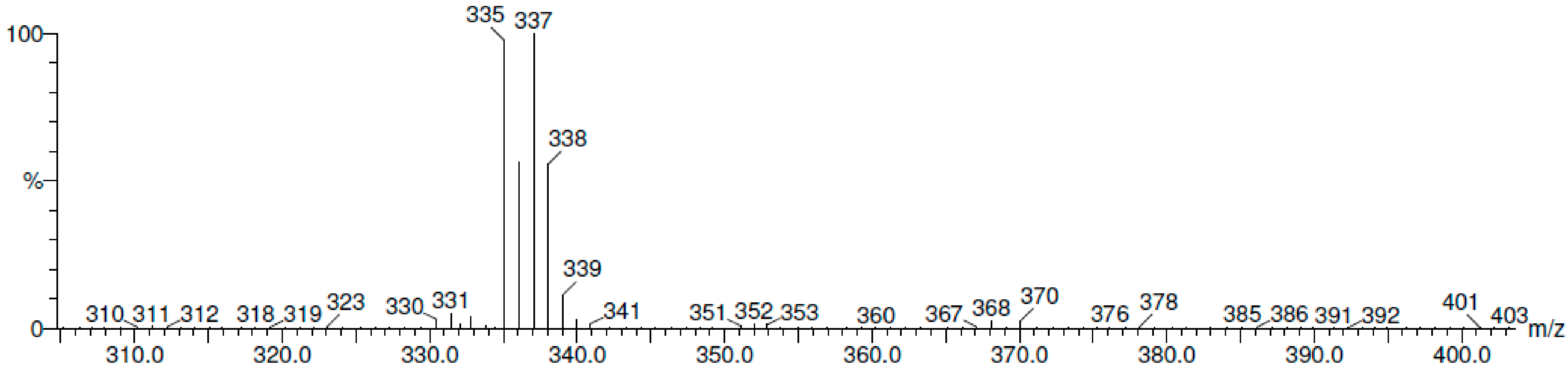

Minimum:				-1.5			
Maximum:		5.0	10.0	50.0			
Mass	Calc. Mass	mDa	PPM	DBE	i-FIT	i-FIT (Norm)	Formula
337.0483	337.0487	-0.4	-1.2	8.5	37.5	2.3	C14 H16 N4 O 81Br
	337.0474	0.9	2.7	3.5	37.6	2.5	C13 H20 O5 81Br
	337.0519	-3.6	-10.7	0.5	37.3	2.2	C3 H16 N10 O4 81Br
	337.0447	3.6	10.7	4.5	36.7	1.5	C9 H16 N6 O3 81Br
	337.0532	-4.9	-14.5	5.5	36.1	1.0	C4 H12 N14 81Br
	337.0433	5.0	14.8	-0.5	37.2	2.1	C8 H20 N2 O7 81Br

Figure S8: ^{1}H NMR (300 MHz, MeOD) spectrum for 6-bromo-8,1'-dihydro-isoplysin A (**2**)

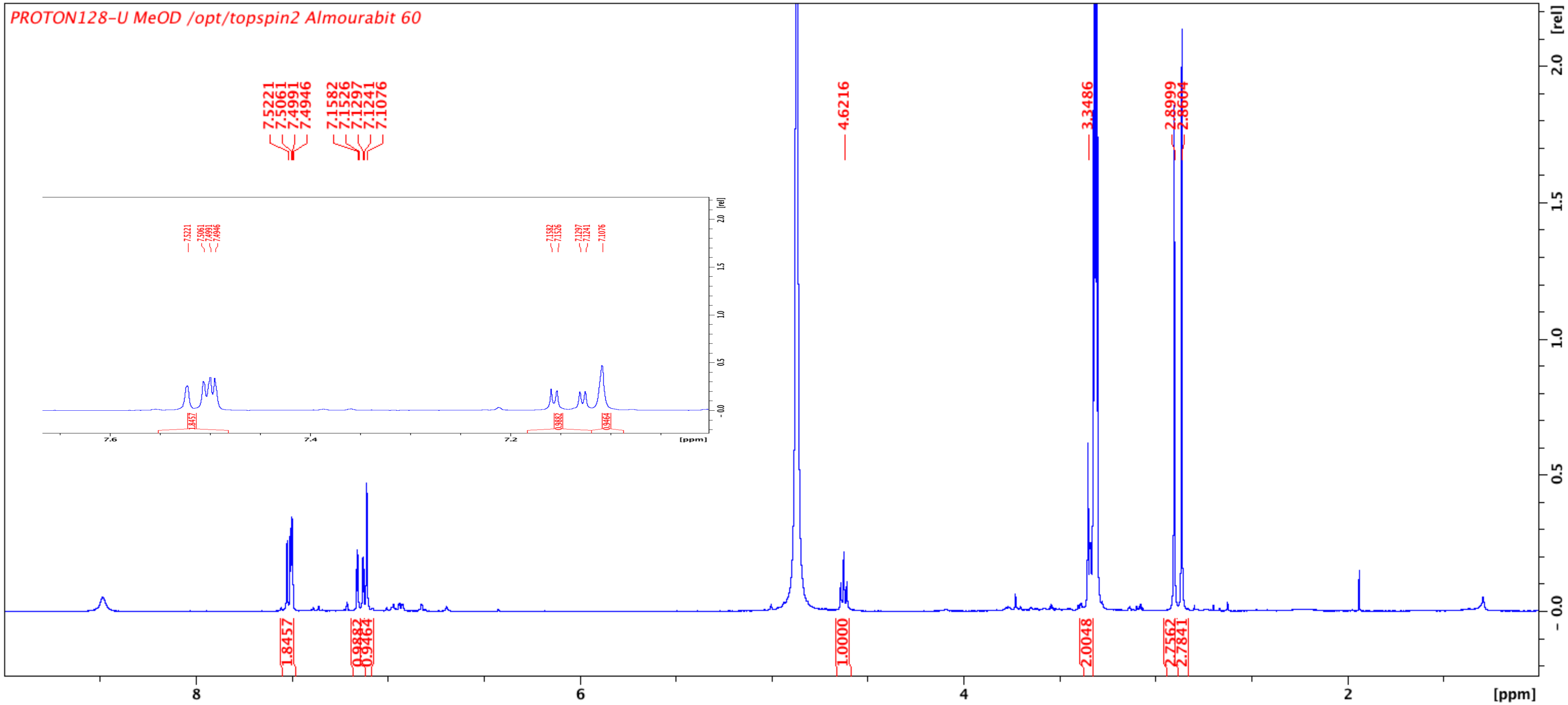

Figure S9: ^{13}C NMR (300 MHz, MeOD) spectrum for 6-bromo-8,1'-dihydro-isoplysin A (**2**)

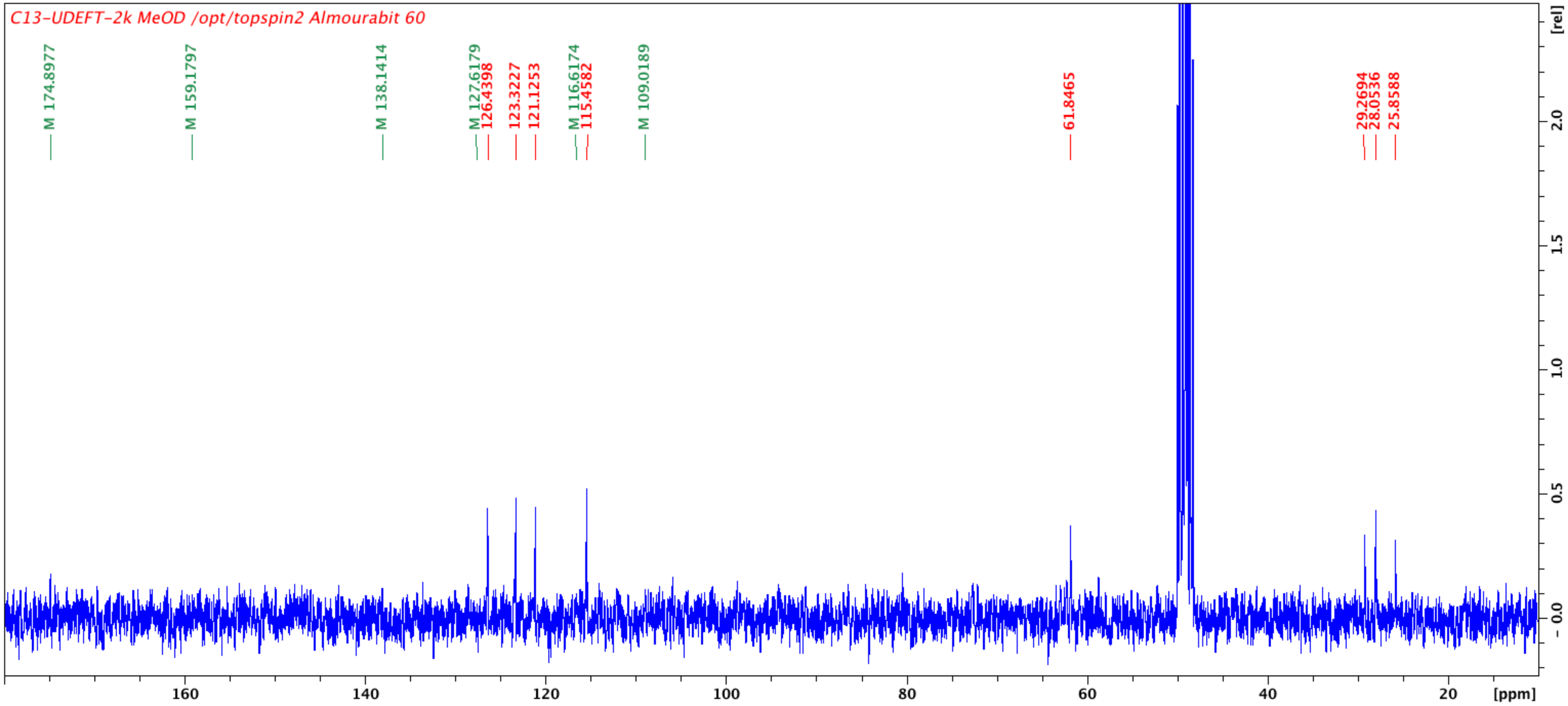

Figure S10: ^{1}H-^{1}H COSY NMR (300 MHz, MeOD) spectrum for 6-bromo-8,1'-dihydro-isoplysin A (**2**)

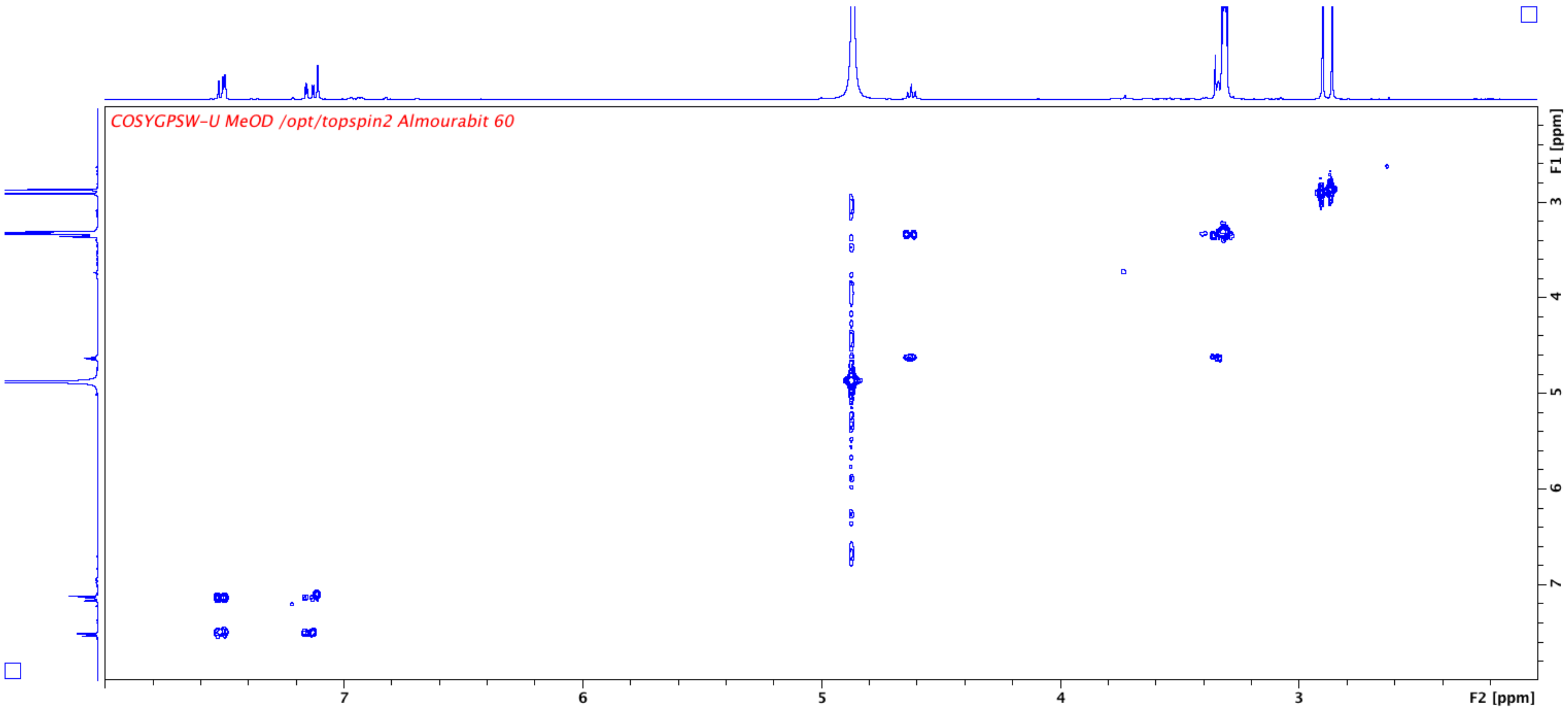

Figure S11: HSQC NMR (300 MHz, MeOD) spectrum for 6-bromo-8,1’-dihydro-isoplysin A (**2**)

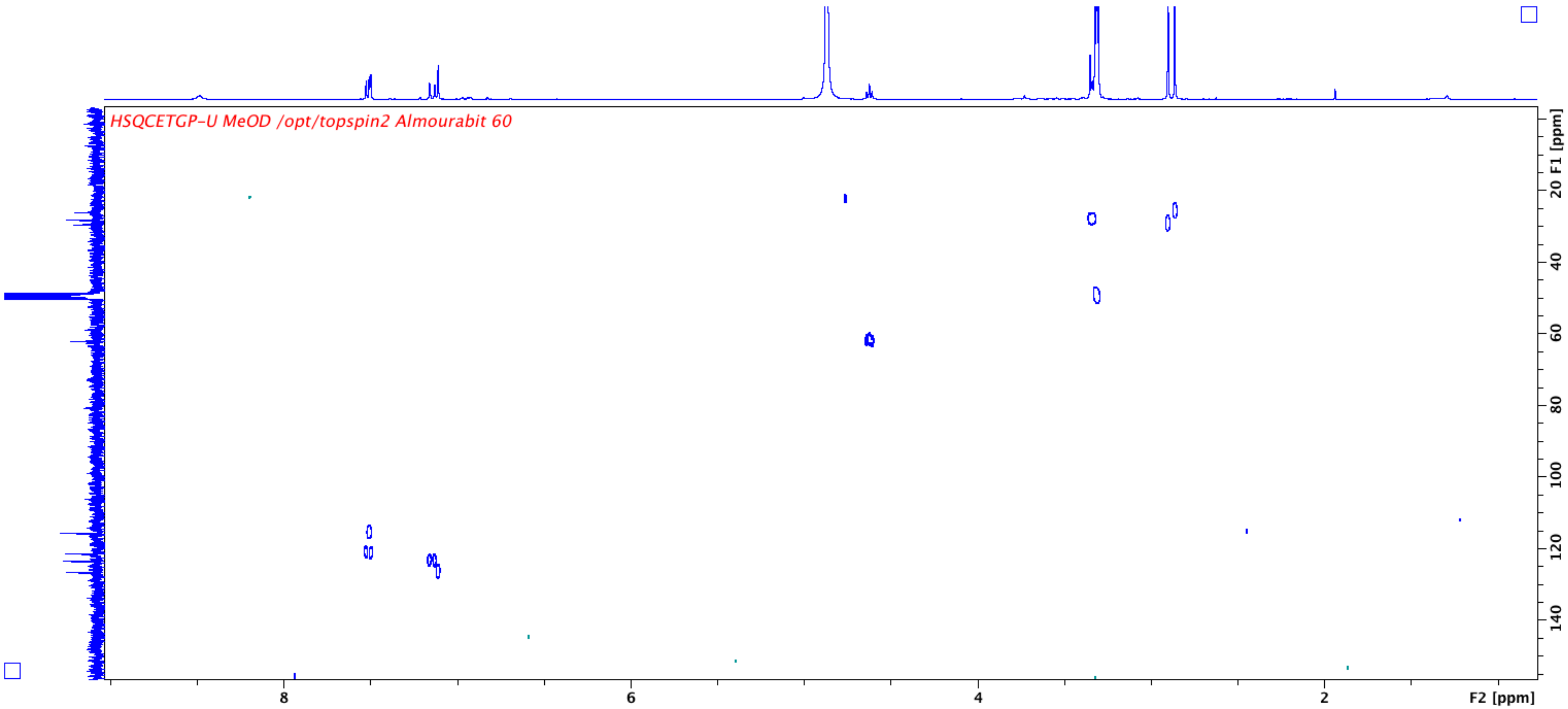

Figure S12: ^{1}H-^{13}C HMBC NMR (300 MHz, MeOD) spectrum for 6-bromo-8,1'-dihydro-isoplysin A (**2**)

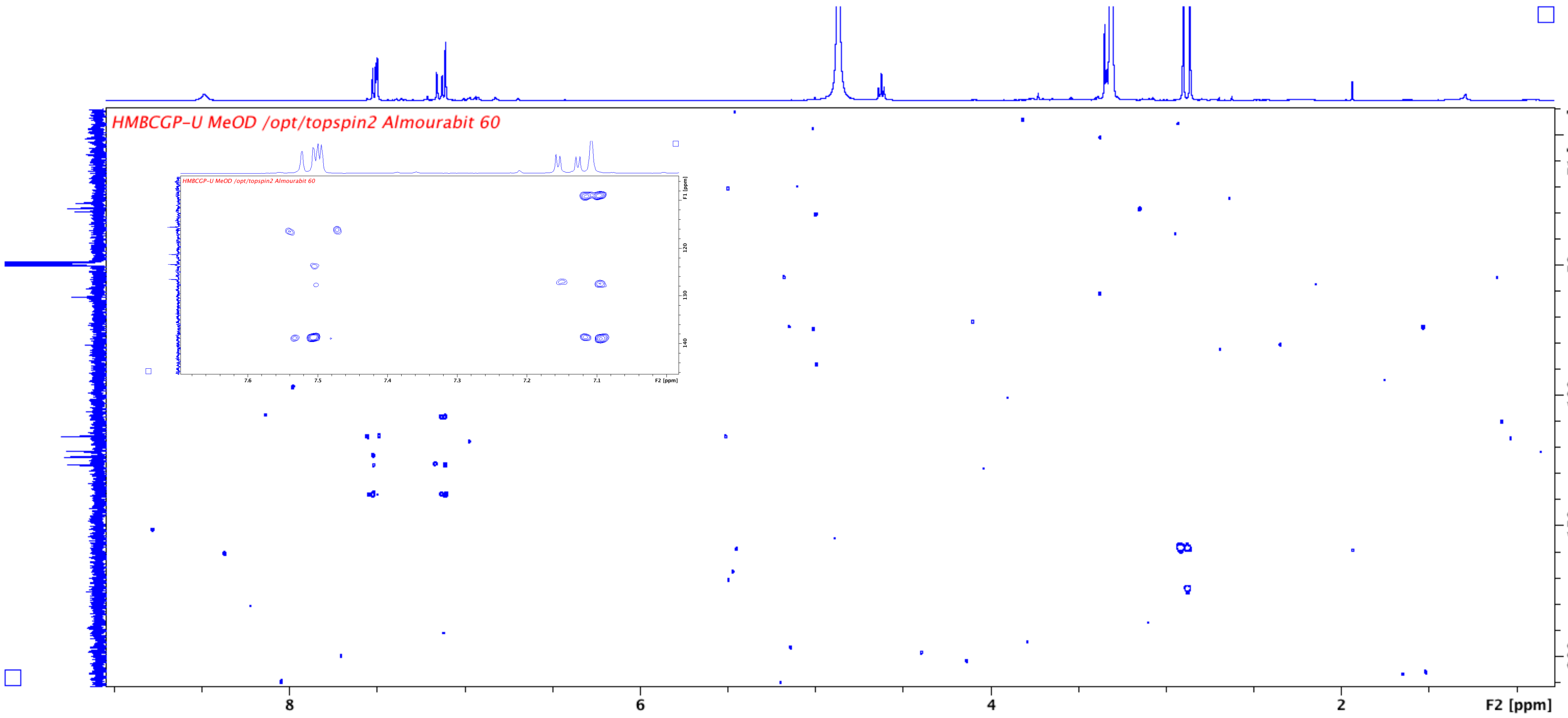

Figure S13: HRMS spectrum for 5,6-dibromo-8,1'-dihydro-isoplysin A (**3**)

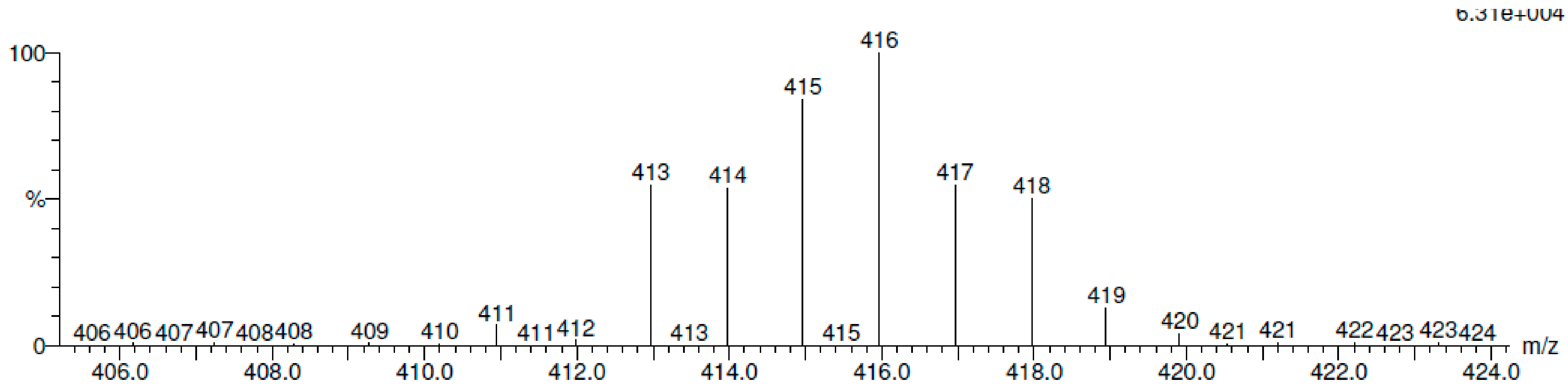

Minimum:				-1.5			
Maximum:		15.0	10.0	50.0			
Mass	Calc. Mass	mDa	PPM	DBE	i-FIT	i-FIT (Norm)	Formula
414.9630	414.9520	11.0	26.5	12.5	41.0	3.3	C20 H15 79Br 81Br
	414.9731	-10.1	-24.3	7.5	40.9	3.2	C17 H19 O2 79Br 81Br
	414.9592	3.8	9.2	8.5	40.7	3.0	C14 H15 N4 O 79Br 81Br

Figure S14: ^{1}H NMR (600 MHz, MeOD) spectrum for 5,6-dibromo-8,1'-dihydro-isoplysin A (**3**)

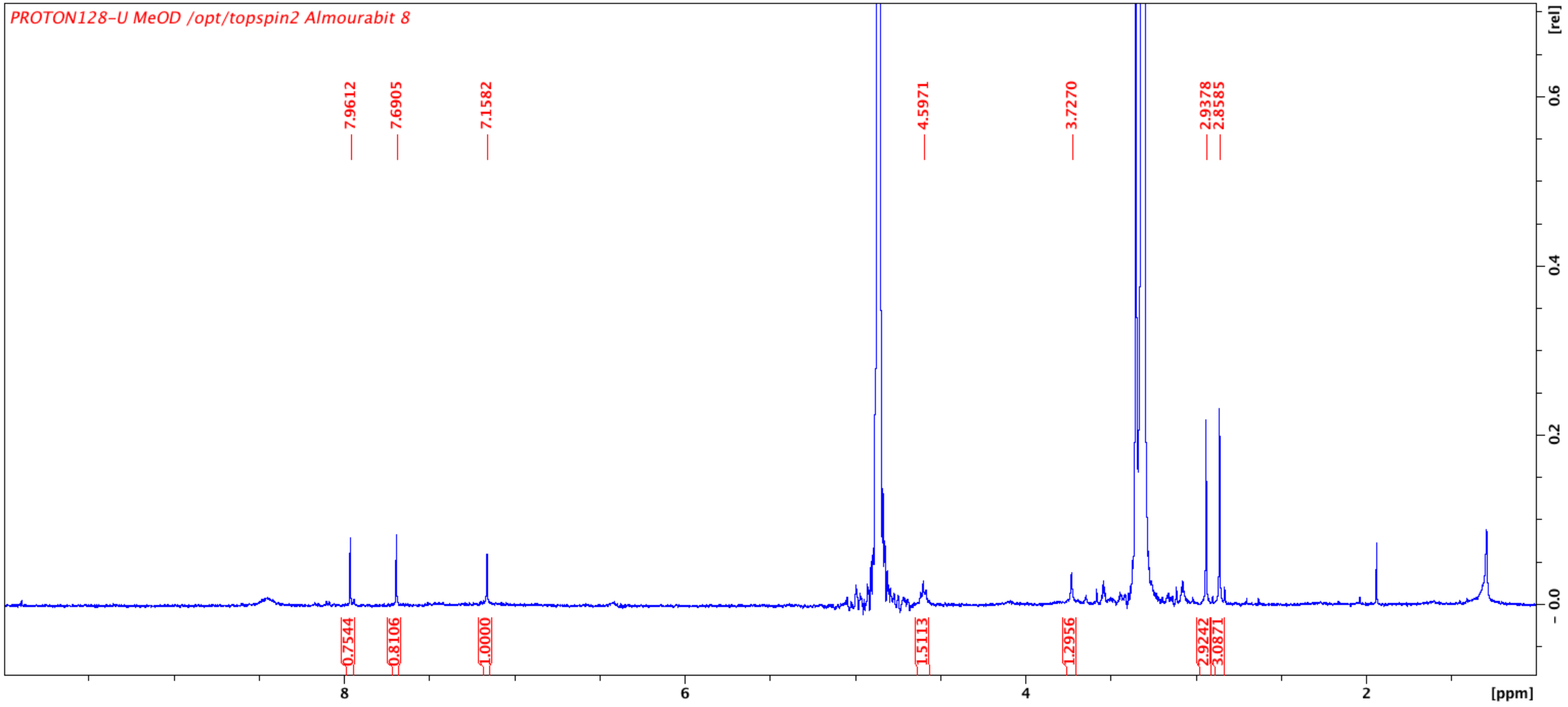

Figure S15: ^{13}C NMR (600 MHz, MeOD) spectrum for 5,6-dibromo-8,1'-dihydro-isoplysin A (**3**)

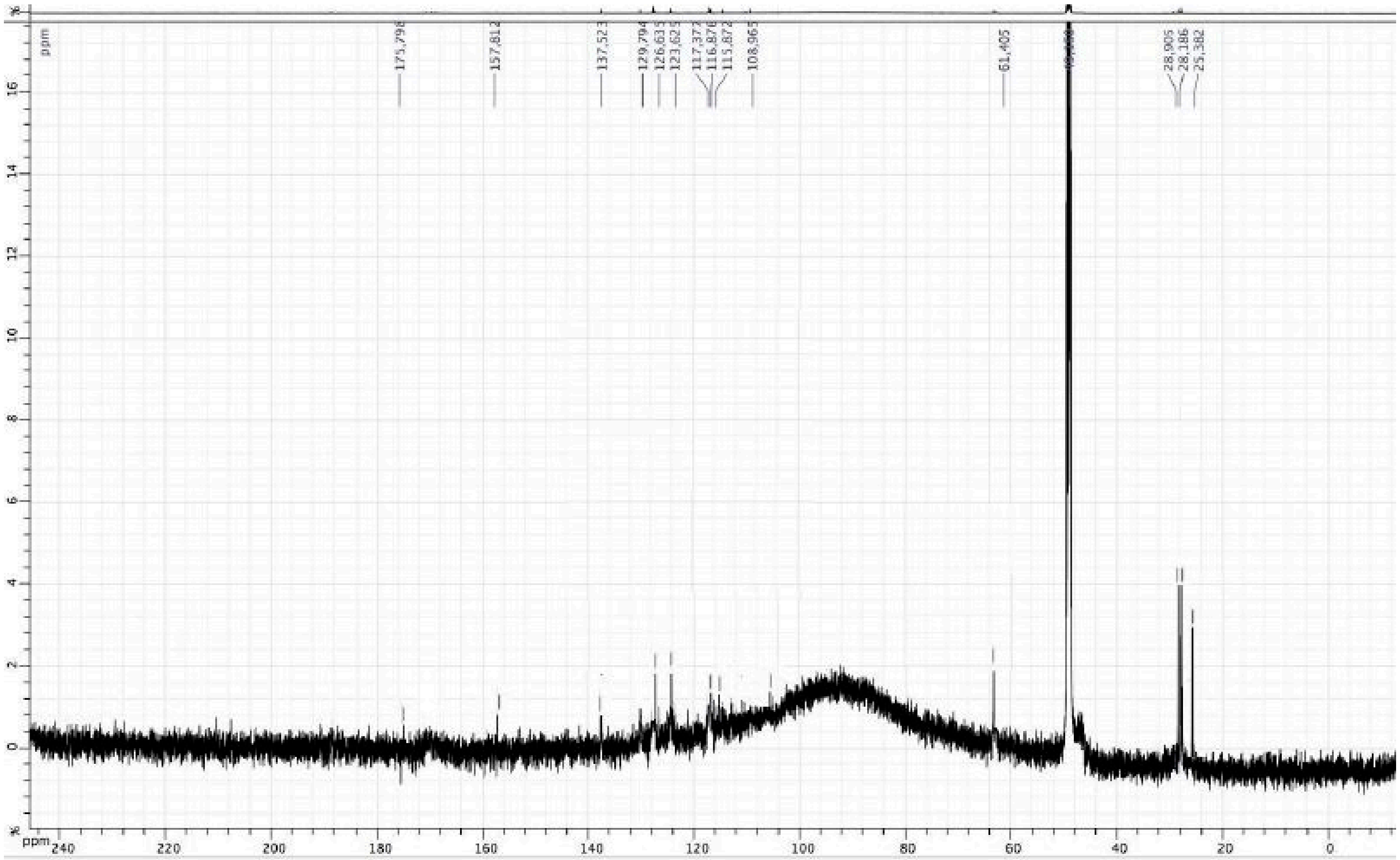

Figure S16: ^{1}H NMR (600 MHz, MeOD) spectrum for 8-oxo-tryptamine (**4**)

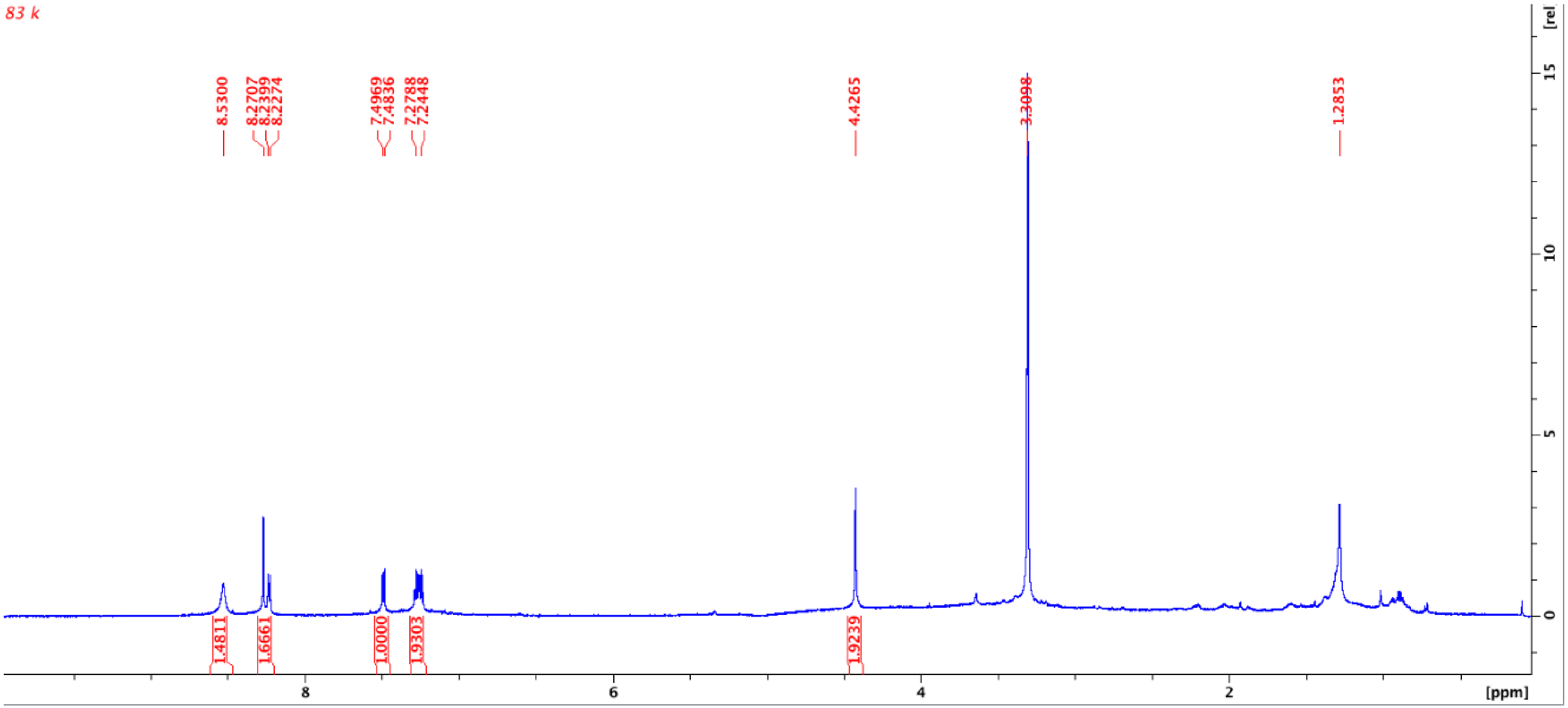

Figure S17: ^{13}C NMR (600 MHz, MeOD) spectrum for 8-oxo-tryptamine (**4**)

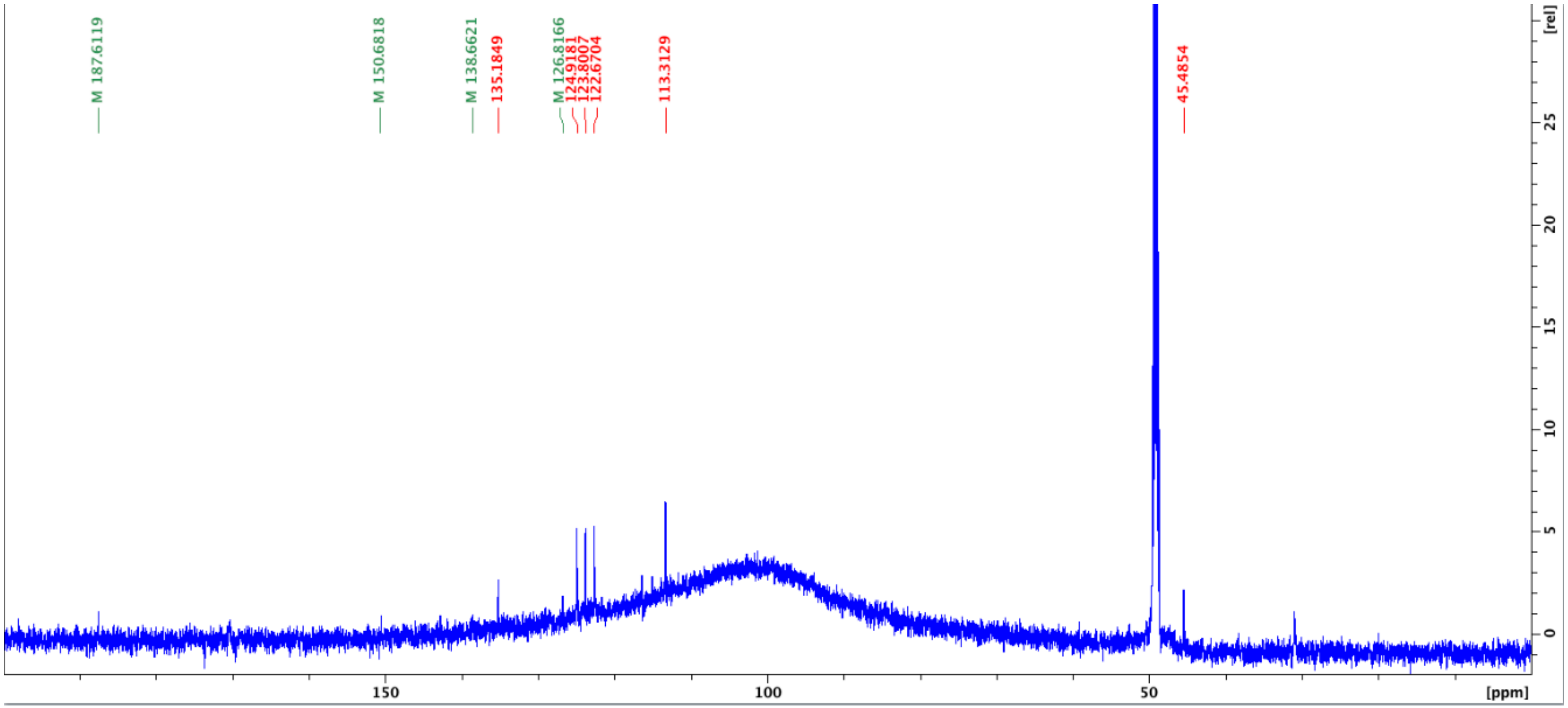

Figure S18: ^{1}H NMR (300 MHz, MeOD) spectrum for tryptamine (**5**)

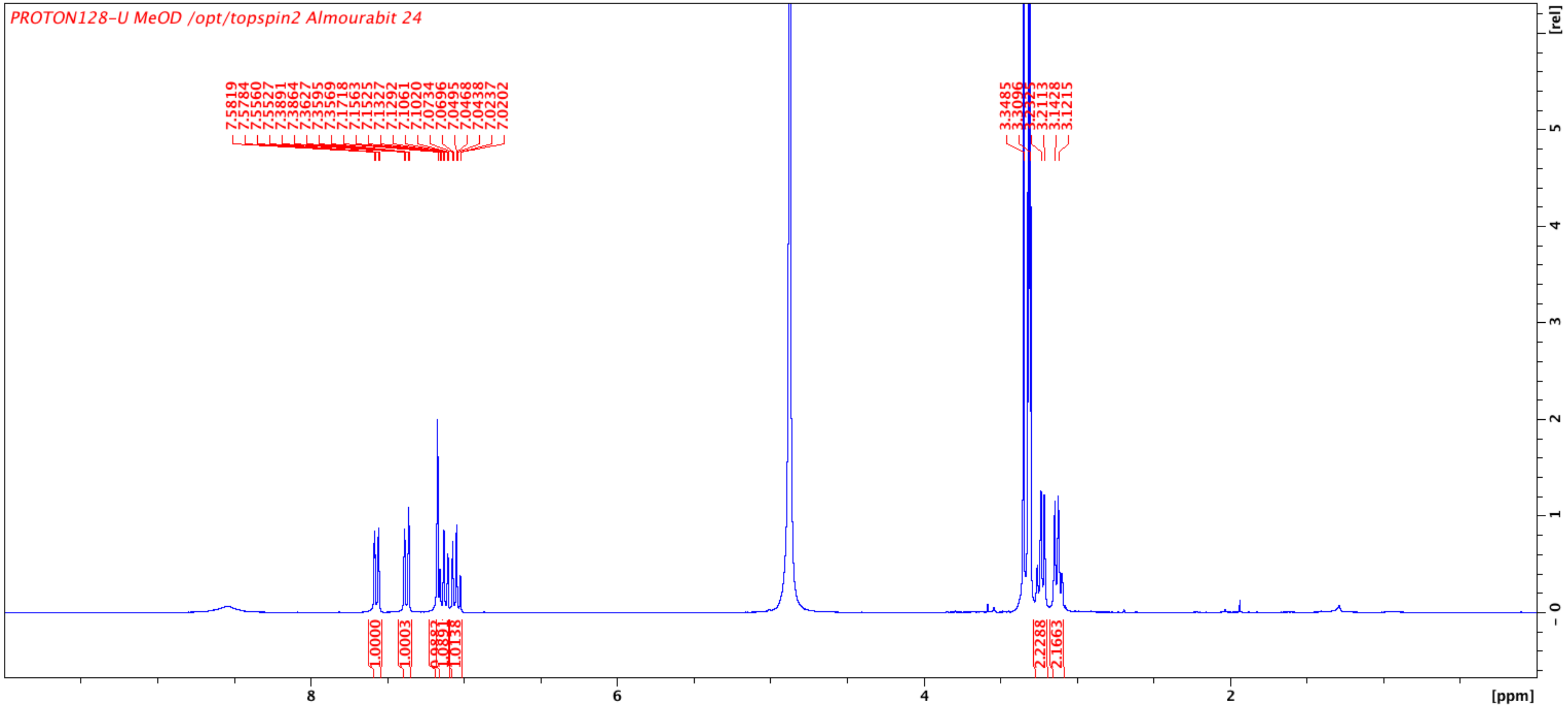

Figure S19: ^{13}C NMR (300 MHz, MeOD) spectrum for tryptamine (**5**)

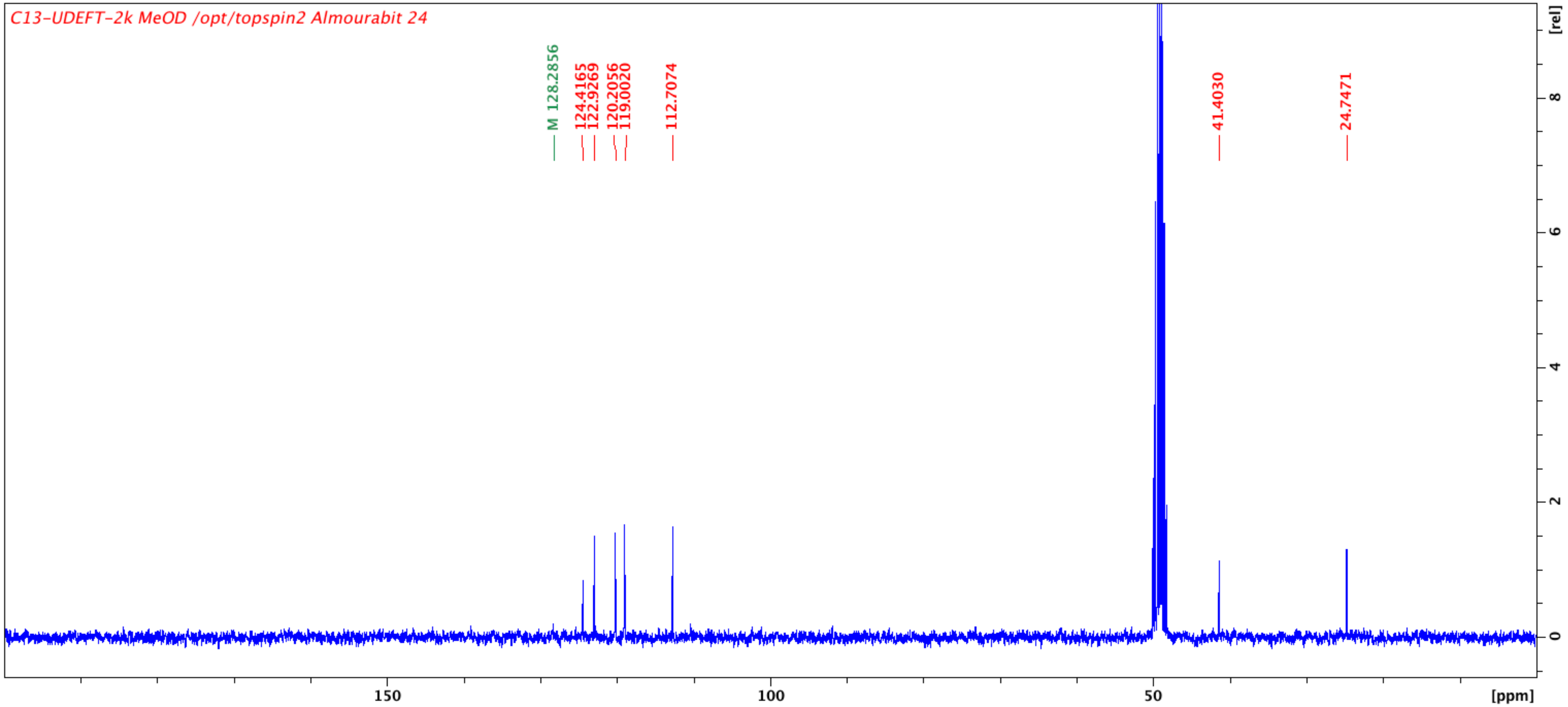

Figure S20: ^{1}H NMR (500 MHz, DMSO) spectrum for (*E*)-6-bromo-2'-demethyl-3'-*N*-methylaplysinopsine (**6**) and (*Z*)-6-bromo-2'-demethyl-3'-*N*-methylaplysinopsine (**7**)

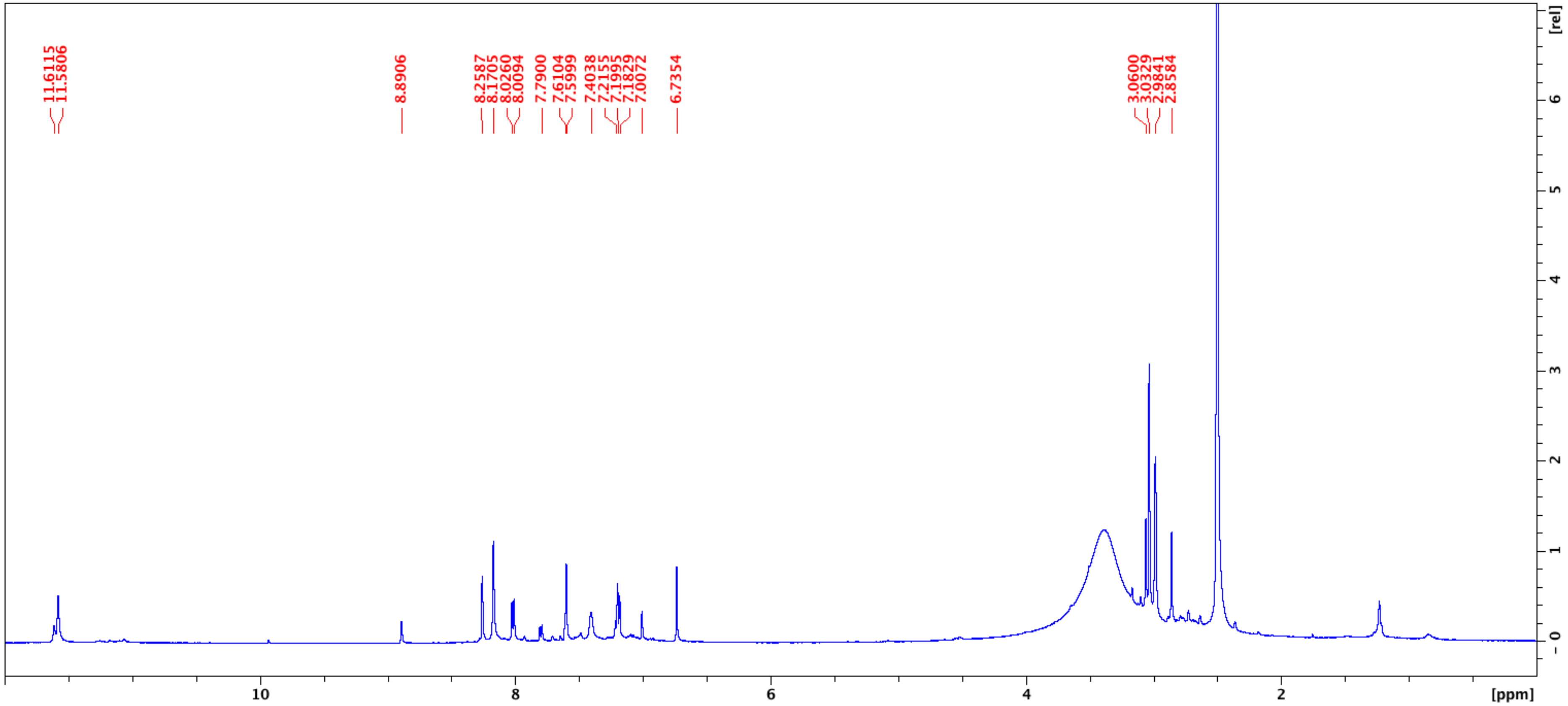

Figure S21: ^{13}C NMR (500 MHz, MeOD) spectrum for (*E*)-6-bromo-2'-demethyl-3'-*N*-methylaplysinopsine (**6**) and (*Z*)-6-bromo-2'-demethyl-3'-*N*-methylaplysinopsine (**7**)

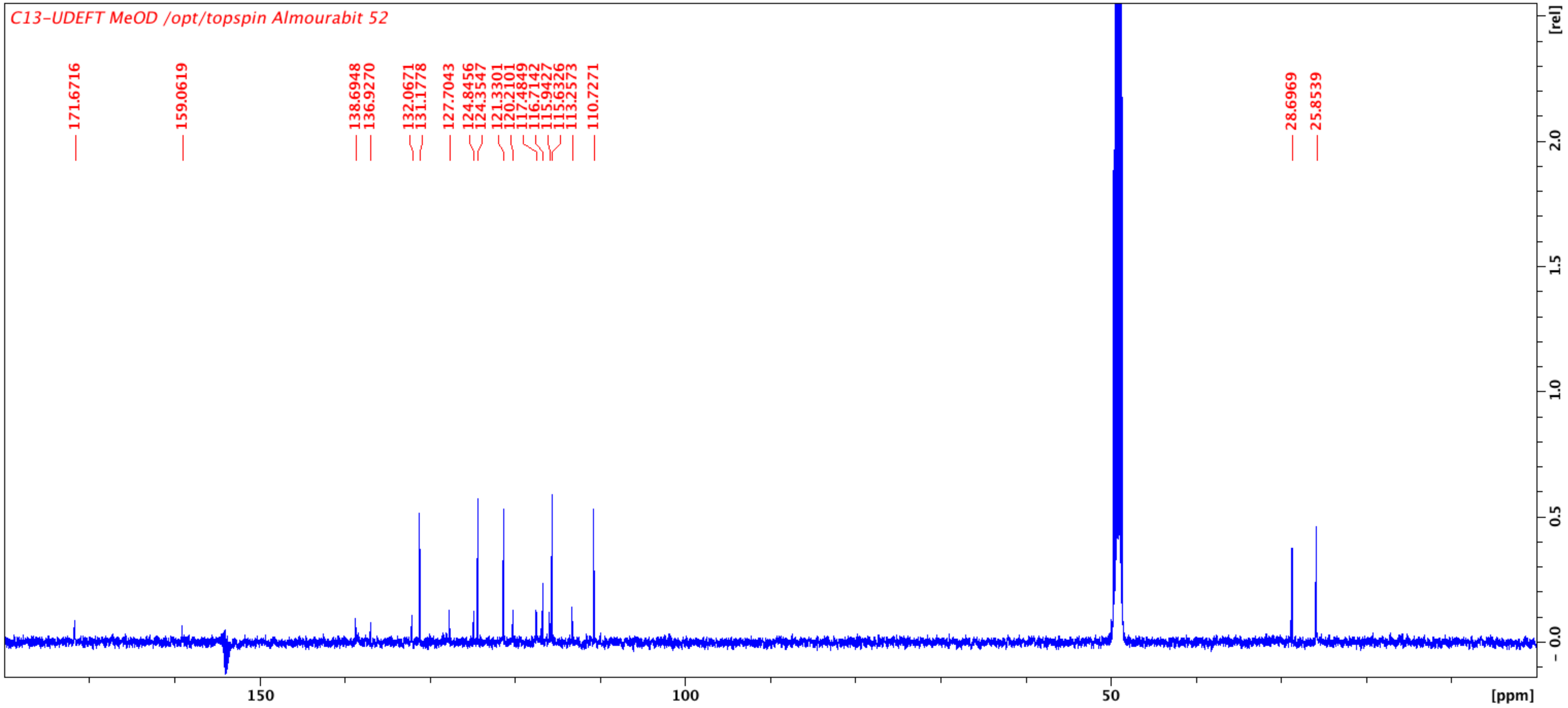

Supplement: Supplementary file 1 [file marinedrugs-17-00167-s001.pdf]
